# Using aircraft location data to estimate current economic activity

**DOI:** 10.1038/s41598-020-63734-w

**Published:** 2020-05-05

**Authors:** Sam Miller, Helen Susannah Moat, Tobias Preis

**Affiliations:** 1 Data Science Lab, Behavioural Science, Warwick Business School, University of Warwick, Scarman Road, Coventry, CV4 7AL UK; 2 The Alan Turing Institute, British Library, 96 Euston Road, London, NW1 2DB UK

**Keywords:** Statistics, Scientific data

## Abstract

Aviation is a key sector of the economy, contributing at least 3% to gross domestic product (GDP) in the UK and the US. Currently, airline performance statistics are published with a three month delay. However, aircraft now broadcast their location in real-time using the *Automated Dependent Surveillance Broadcast* system (ADS-B). In this paper, we analyse a global dataset of flights since July 2016. We first show that it is possible to accurately estimate airline flight volumes using ADS-B data, which is available immediately. Next, we demonstrate that real-time knowledge of flight volumes can be a leading indicator for aviation’s direct contribution to GDP in both the UK and the US. Using ADS-B data could therefore help move us towards real-time estimates of GDP, which would equip policymakers with the information to respond to shocks more quickly.

## Introduction

Most economic statistics, such as GDP, are released with a significant delay. Estimating their current values before they are published is known as “nowcasting”^1^. Previous studies have used real-time internet data, from sources such as *Google*^2–6^, *Twitter*^7,8^ and *Wikipedia*^9,10^, to nowcast economic data. Better nowcasts are highly valuable, as major economic policy tools such as interest rates can take up to 20 months to fully impact the economy^11^. When faced with shocks like the 2008 financial crisis, policymakers must therefore respond as quickly as possible, which requires accurate knowledge of the current state of the economy. Failure to do so has deepened past recessions^12^, leading to political instability across Europe following both the Great Depression and the 2008 financial crisis.

Aviation is a key economic sector, contributing at least 3% to GDP in the UK and the US^13,14^. Aircraft now broadcast their location, among other data, in real-time using the Automated Dependent Surveillance Broadcast (ADS-B) system. So far, ADS-B data has rarely been used outside its intended application in the aviation sector. An exception is one study that showed that ADS-B data could track corporate jets, therefore providing a leading indicator for certain mergers between firms^15^. The current paper analyses whether ADS-B data can help nowcast airline performance and aviation’s direct contribution to GDP. In contrast to real-time ADS-B data, the current statistics for these variables are published with a three month delay because they require surveys of businesses^16–19^. Faster statistics could help policymakers respond more quickly to future economic shocks, thereby limiting their damage.

## Methods

### ADS-B data

We retrieve aircraft data from the *ADS-B Exchange*^20^. Commercial aircraft in Europe have been required to broadcast ADS-B data since 2017^21^, and it has been mandatory for US aircraft since January 2020^22^. These broadcasts include the aircraft’s speed and location, specified as their altitude, latitude and longitude alongside a timestamp. Each ADS-B message also includes a six-digit hex identification code assigned to the aircraft by the *International Civil Aviation Organisation* (ICAO). Amongst other things, the ICAO code makes it possible to link an aircraft to its operating airline by looking up the ICAO code in a corresponding database. This pre-processing is carried out by *ADS-B Exchange* and the operating airline is included in each of the resulting ADS-B records.

**Figure 1:**
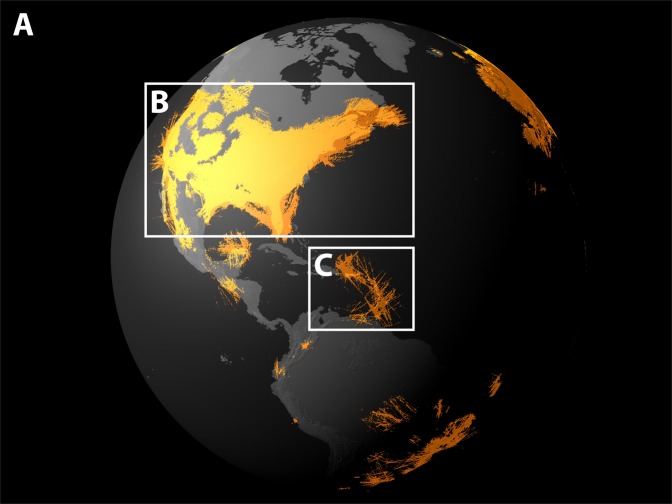
Recorded flight paths over the western hemisphere on 30th September 2016. (**A**) In orange, we depict the locations from which at least one ADS-B message was received by the network of receivers. (**B**) The network covers large parts of the United States and in particular their coastal regions. Visual inspection of the US east coast reveals that the land-based receivers are capable of tracking air traffic over coastal waters too. (**C**) In regions within coverage but with less dense air traffic flow, e.g. in the broad vicinity of the Caribbean Sea, distinct flight routes emerge. The increase in coverage from 2016 to 2018 is shown in Supplementary Fig. S1. The base layer of this map utilises the ALOS World 3D global digital surface model provided by the Japan Aerospace Exploration Agency (JAXA), which is available to use with no charge via https://www.eorc.jaxa.jp/ALOS/en/ (JAXA).

ADS-B messages are unencrypted, in order to be receivable by other aircraft, which means they are available to anyone with an ADS-B receiver. The *ADS-B Exchange* collects data from thousands of receivers^20^. The resulting database covers global flight activity (Fig. 1). We analyse the period from July 2016 to December 2018. We note that coverage has improved over time, as the number of receivers feeding the database has grown (see Supplementary Fig. S1).

**Figure 2:**
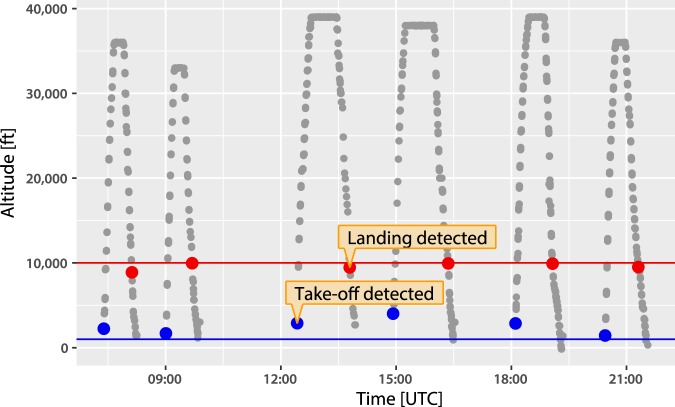
The flight-counting algorithm. An example of the flight-counting algorithm crawling through altitude data from real-time ADS-B messages. The algorithm identifies a take-off and landing of six separate flights for this aircraft over the course of a day. These form a new data structure, where we have one row for each flight rather than one row for each ADS-B observation. In total we identify 67 million separate flights from July 2016 to December 2018.

The raw data we analyse contains roughly 25 billion messages. First, we reduce this data from one row for each message to one row for each flight. Figure 2 shows how we identify take-offs and landings by analysing the altitude of an aircraft over time (see also Supplementary Figs. S2 to S6). Applying this method, we extract 67 million separate flights and record their take-off and landing time as well as corresponding locations. Next, we aggregate these flights by month and airline to generate estimates for published airline statistics. This further reduces our dataset to 303 monthly airline flight counts for the UK, and 405 for the US. We include the largest 13 UK and 15 US airlines, with the cut-off for airline inclusion set at 1% of total air traffic in each country. Overall, our ADS-B data captures 83% of UK flights and 41% of US flights since July 2016.

### Published aviation statistics

**Figure 3:**
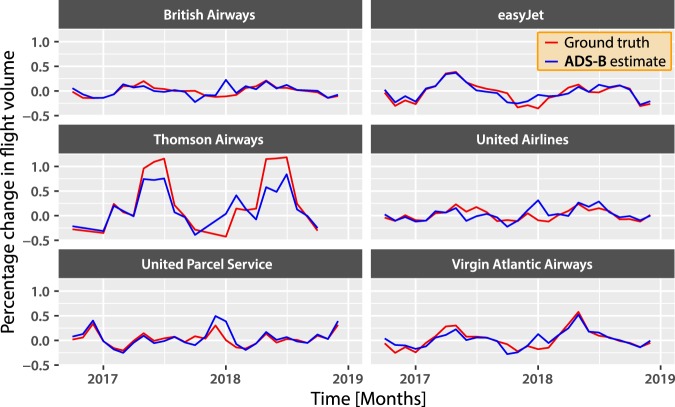
Comparing official aviation statistics to measures derived from ADS-B data. Plots of the percentage change in official flight count and ADS-B flight count for six UK and US airlines. The ADS-B estimate of the monthly change tracks the official statistics very closely, although there is some variation in accuracy across airlines. We suggest the two most likely sources of error are the database that maps aircraft to airline, and imperfect coverage of the ADS-B data.

Both the UK^16^ and US^18^ aviation authorities publish monthly airline statistics. They contain a range of performance indicators, such as flight volume and capacity utilisation, but are currently released with a three month delay. Figure 3 depicts the monthly percentage change in both the airline statistics and in flight volumes calculated using the ADS-B data. Visual inspection suggests there is a strong correlation. However, there is clear seasonality for both countries (see Supplementary Figs. S7 and S8). We account for this seasonality in our later analysis.

Finally, we collect economic data from the *UK Office for National Statistics* (ONS)^17^ and *US Bureau of Economic Analysis* (BEA)^19^. Both the ONS and BEA publish a GDP series that is split by industry, from which we consider air transport. The UK series is a monthly time series dating back to 1997, and the US series is a quarterly time series dating back to 2005. When analysing these series, we consider key time series properties, such as stationarity, to avoid drawing misleading conclusions (see Supplementary Figs. S9 and S10).

## Results

### Estimating airline flight volume

For each airline, we aim to generate rapid estimates of flight volume across time. Some airlines are much larger than others. To ensure comparability between airlines, we therefore normalise the flight volume data by indexing the first period to each airline for 100. We then re-scale subsequent periods so they are measured relative to the first. An airline whose original flight counts were (5000, 6000, … 6500) would be normalised to (100, 120, … 130). A normalised flight volume of 120 reflects a flight volume 20% higher than the first period.

A reasonable baseline model would be an autoregressive (AR) model where we estimate normalised airline flight volumes with their own history:

1 $${y}_{i,t}={\alpha }_{i}+{\gamma }_{t}+\beta {y}_{i,t-3}+{\varepsilon }_{i,t},$$

where ${y}_{i,t}$ is the number of flights and ${\varepsilon }_{i,t}$ is a noise term for airline *i* in month *t*. Due to the three month publication lag for the official flight volume statistics, when nowcasting the flight volume for month *t* we only have official data from month *t*−3. The baseline therefore includes an AR(3) term, ${y}_{i,t-3}$ and $\beta$ is the weight on the AR(3) term.

We also derive binary (“dummy”) variables from the longitudinal data structure. ${\gamma }_{t}$ are coefficients for dummy variables for each month (12 in total), which proxy for seasonality. A positive value for ${\gamma }_{t}$ would reflect that flight volumes are usually higher than average in month *t*. ${\alpha }_{i}$ are coefficients for 28 airline-specific dummy variables, which capture the airline’s average growth over time. A positive value of ${\alpha }_{i}$ would reflect an increase in the mean flight volume for airline *i* across the time period.

To measure the performance boost from ADS-B data, we add this data to the baseline model. Denoting ${x}_{i,t}$ as the ADS-B flight count for airline *i* in period *t*, and *δ* as the weight on the ADS-B term:

2 $${y}_{i,t}={\alpha }_{i}+{\gamma }_{t}+\delta {x}_{i,t}+\beta {y}_{i,t-3}+{\varepsilon }_{i,t}$$

Table 1 shows that adding ADS-B data boosts the in-sample accuracy of all baseline models, regardless of whether month and airline dummies are included (see Supplementary Table S1 for similar results when the model includes month dummies or airline dummies alone). This shows that ADS-B data can help estimate dynamic airline-specific changes in flight volume, and does not only proxy seasonality or differences in airline growth.

**Table 1:** Estimating airline flight volume: in-sample results.

Model	Simple	Complex	Simple	Complex
	UK		US	
Baseline adj *R*^2^	0.06	0.66	0.36	0.75
ADS-B adj *R*^2^	0.54	0.90	0.56	0.89
Number of parameters	2	27	2	29
Number of airlines	13	13	15	15

In-sample adjusted *R*^2^ scores from models built to generate rapid estimates of airline flight volume. All models are unpenalised linear regressions. The baseline model is autoregressive: it estimates the change in each airline’s monthly flights using the most recently available flight count statistics. The ADS-B model additionally includes the ADS-B estimate of each airline’s monthly flights as a predictor. The simple model does not include any further predictors. The complex model includes binary variables for each airline and month, to capture seasonality and differences in airline growth across the period. ADS-B data boosts performance across all model specifications, including against the more complex baseline. This shows that ADS-B data can help estimate dynamic airline-specific changes in flight volume, and does not only proxy seasonality or differences in airline growth.

Our results so far suggest that ADS-B improves in-sample estimates of airline flight volume. However, in-sample scores may overstate true predictive accuracy. To assess out-of-sample performance, we use one-step-ahead adaptive nowcasting^23^, originally developed to help generate rapid indicators of flu incidence using *Google* search volume. For each month *t* in our dataset, we train the model with data from months $\in \mathrm{[1,}t-\mathrm{1]}$. We penalise the model’s coefficients, to avoid overfitting, using LASSO regression with 5-fold cross-validation (see Supplementary Methods for regularising out-of-sample forecasts). The penalised model then estimates the test month *t*. We record the estimate for each airline, and use the mean absolute error (MAE) as the score for month *t*. As we move through the analysis period, thereby increasing *t*, we re-fit the model to add new training data (hence “adaptive”). This procedure only uses past data to predict the present, so we know performance is tested out-of-sample.

For both the UK and US, we construct a baseline adaptive nowcasting model which produces estimates for each airline. The baseline adaptive nowcasting model contains airline dummies (see Supplementary Table S2 for model performance with different dummy configurations). The ADS-B model adds ADS-B data to the baseline adaptive nowcasting model.

**Figure 4:**
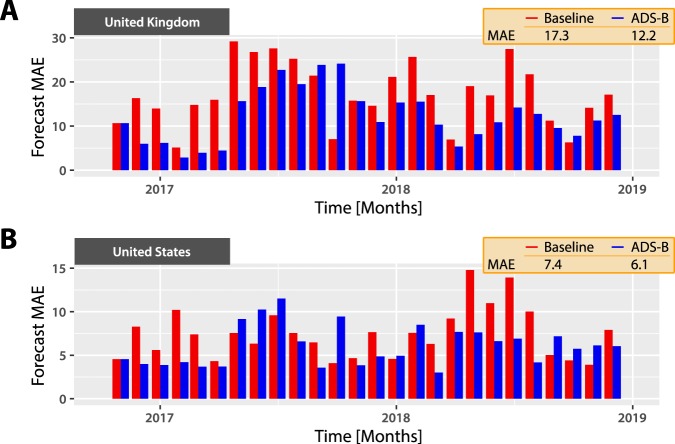
Adaptive nowcasting of airline flight volume. We build adaptive nowcasting models^23^ to generate rapid estimates of flight volume for UK and US airlines. We investigate whether models enhanced with real-time ADS-B data deliver more accurate estimates than nowcasting models based on historic flight volume alone. For each month $t\in \mathrm{[5,}T]$, we fit a model with all data up to $t-1$, then test performance in month *t*. The model is an autoregressive linear regression penalised using LASSO and tuned through 5-fold cross-validation. (**A**) Performance for the UK. (**B**) Performance for the US. The red series show the mean absolute error (MAE) from the baseline model with no ADS-B data, whereas the blue series show the MAE when adding ADS-B data. We find that including ADS-B data as a predictor improves rapid estimates of airline flight volume in both the UK and the US.

Figure 4 depicts our adaptive nowcasting results. Adding ADS-B data reduces the MAE by 29% for the UK (baseline MAE = 17.3, ADS-B MAE = 12.2) and 18% for the US (baseline MAE = 7.4, ADS-B MAE = 6.1). We also show the distribution of errors for each model over time in Supplementary Fig. S11. These results hold across a range of dummy specifications and training windows (see Supplementary Tables S2 and S3). Therefore ADS-B data also improves out-of-sample estimates of airline flight volume.

#### Estimating economic activity

We next analyse whether ADS-B data may help estimate aviation’s direct contribution to GDP. Both the UK and US aviation GDP series are non-stationary based on Augmented Dickey-Fuller tests (UK: *Dickey-Fuller* = −1.4; US: *Dickey-Fuller* = −2.9; both *p*s > 0.05). Therefore their distributions are not constant over time, so we cannot use them for regression. Instead, we use the rolling annual percentage change in GDP, which deals effectively with both non-stationarity and seasonality (see Supplementary Figs. S7 to S10).

Our baseline specification for the annual percentage change in aviation’s direct contribution to GDP $\Delta {z}_{t}$ is

3 $$\Delta {z}_{t}=\alpha +\beta \Delta {z}_{t-j}+{\varepsilon }_{t},$$

where ${\varepsilon }_{t}$ is a noise term. There is a two month publication lag for the first complete estimate of UK GDP, and a one quarter lag for the US. Therefore $\Delta {z}_{t-j}$ is the most recent value known at month *t*, where $j=2$ for the UK and $j=1$ for the US. The augmented model includes the rolling annual percentage change in ADS-B flight volume $\Delta {x}_{t}$:

4 $$\Delta {z}_{t}=\alpha +\beta \Delta {z}_{t-j}+\gamma \Delta {x}_{t}+{\varepsilon }_{t}$$

The in-sample results are promising: adding ADS-B data boosts adjusted ${R}^{2}$ from 31% to 55% for the UK and 12% to 42% for the US. However, there are only 18 monthly periods for the UK and 6 quarterly periods for the US due to the limited time series of ADS-B data. These sample sizes are clearly too small to assess out-of-sample performance with an adaptive nowcasting model.

We previously showed that ADS-B data could help estimate official flight volumes. To obtain greater insight into whether ADS-B data can improve nowcasts of aviation’s direct contribution to GDP, we therefore substitute the official airline flight volume series in place of the ADS-B data. The official airline series are available for the full period for which we have aviation GDP data for both the UK (from 1997) and the US (from 2005). We again use adaptive nowcasting, but with fixed training window lengths of 60 months for the UK and 8 quarters for the US (see Supplementary Table S6 for results using other training window lengths).

**Figure 5:**
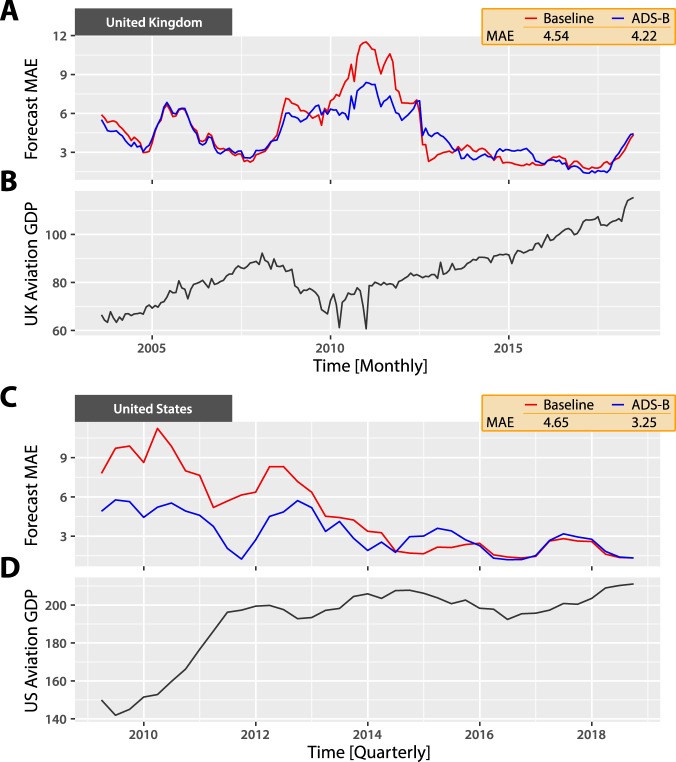
Adaptive nowcasting of aviation’s direct contribution to GDP. We build adaptive nowcasting models [23] to generate rapid estimates of aviation’s direct contribution to GDP in the UK and the US. We investigate whether models enhanced with real-time flight volume data would deliver more accurate estimates than nowcasting models based on historic GDP data alone. Given a training window *w*, for each period $t\in [w,T]$ we fit a model with all data $\in [t-w,t-\mathrm{1]}$, then test performance in period *t*. (**A**) Adding flight volume data reduces the UK nowcast MAE by 7%. (**B**) Aviation’s direct contribution to UK GDP. Visual inspection suggests that flight volume data delivers the greatest improvements in estimates during volatile economic periods, such as the period from 2008 to 2012. (**C**) Similarly, adding flight volume results in the MAE decreasing by 30% in the US. (**D**) Aviation’s direct contribution to GDP in the US. Again, we see that the US model is most improved by flight volume data during the volatile economic period until 2012.

Figure 5 depicts out-of-sample results for GDP estimation. Adding real-time flight volume data reduces the MAE by 7% for the UK (baseline MAE = 4.54, ADS-B MAE = 4.22) and 30% for the US (baseline MAE = 4.65, ADS-B MAE = 3.25). The improvement is greatest during the 2008–2010 financial crisis and the 2012 Euro crisis (see Supplementary Table S5). This may be because the baseline AR model performs worse during volatile economic times.

## Discussion

We have assessed whether ADS-B data can help nowcast aviation statistics, which are currently published with a three month delay. We first show that ADS-B data can accurately estimate airline performance, as measured by their flight volume. Second, we show that real-time knowledge of flight volume is a leading indicator for aviation’s direct contribution to GDP. We find that this indicator is of greatest value during volatile periods, such as the crises between 2008 and 2012. Crisis periods are when rapid estimates for GDP are most crucial for policymakers, as they must take decisions quickly. In certain crises, such as disease outbreaks, real-time information on flight volumes may also be important beyond the economic domain.

The main limitation of our analysis comes from the novelty of ADS-B data. We do not have a long enough time series to determine whether ADS-B data, which we only had access to from July 2016 onward, can directly nowcast GDP out-of-sample. Future work will have access to a longer ADS-B time series and could therefore better evaluate out-of-sample performance. Continued monitoring of ADS-B data will also be important in case its value as an economic indicator changes. For example, airlines who knew ADS-B data were being used to assess their performance may instruct pilots to fly differently. This however seems unlikely given the probable costs of flying more erratically. ADS-B data is therefore likely to be less prone to manipulation than other nowcasting data, such as internet search activity.

Finally, our analysis is restricted to aviation which comprises only 3–5% of GDP in total, including indirect contributions which are not analysed here. However, our methods could be extended to other sectors of the economy where data is shared at a similar level of granularity. As the availability of real-time data increases, we could develop more accurate estimates of a large enough number of economic sectors to build a complete, real-time picture of the economy. In turn, policymakers would be able to respond more effectively to future crises.

## Supplementary information

Supplementary Information.

## Data Availability

This study was a re-analysis of existing datasets that are publicly available at the locations described in the Methods section.
