# Supplementary material for: Using aircraft location data to estimate current economic activity
Source: Sci Rep. 2020 May 5;10:7576. doi: 10.1038/s41598-020-63734-w (PMC7200678; doi:10.1038/s41598-020-63734-w)
Supplement: Supplementary file 1 — Supplementary Information. [file 41598_2020_63734_MOESM1_ESM.pdf]

Supplementary Information

Using aircraft location data to estimate current economic activity

Sam Miller[1,2*], Helen Susannah Moat[1,2] and Tobias Preis[1,2]

[1]Data Science Lab, Warwick Business School
University of Warwick, Scarman Road, Coventry CV4 7AL
[2]The Alan Turing Institute
British Library, 96 Euston Road, London NW1 2DB

*To whom correspondence should be addressed; E-mail: smiller@turing.ac.uk

Supplementary Methods

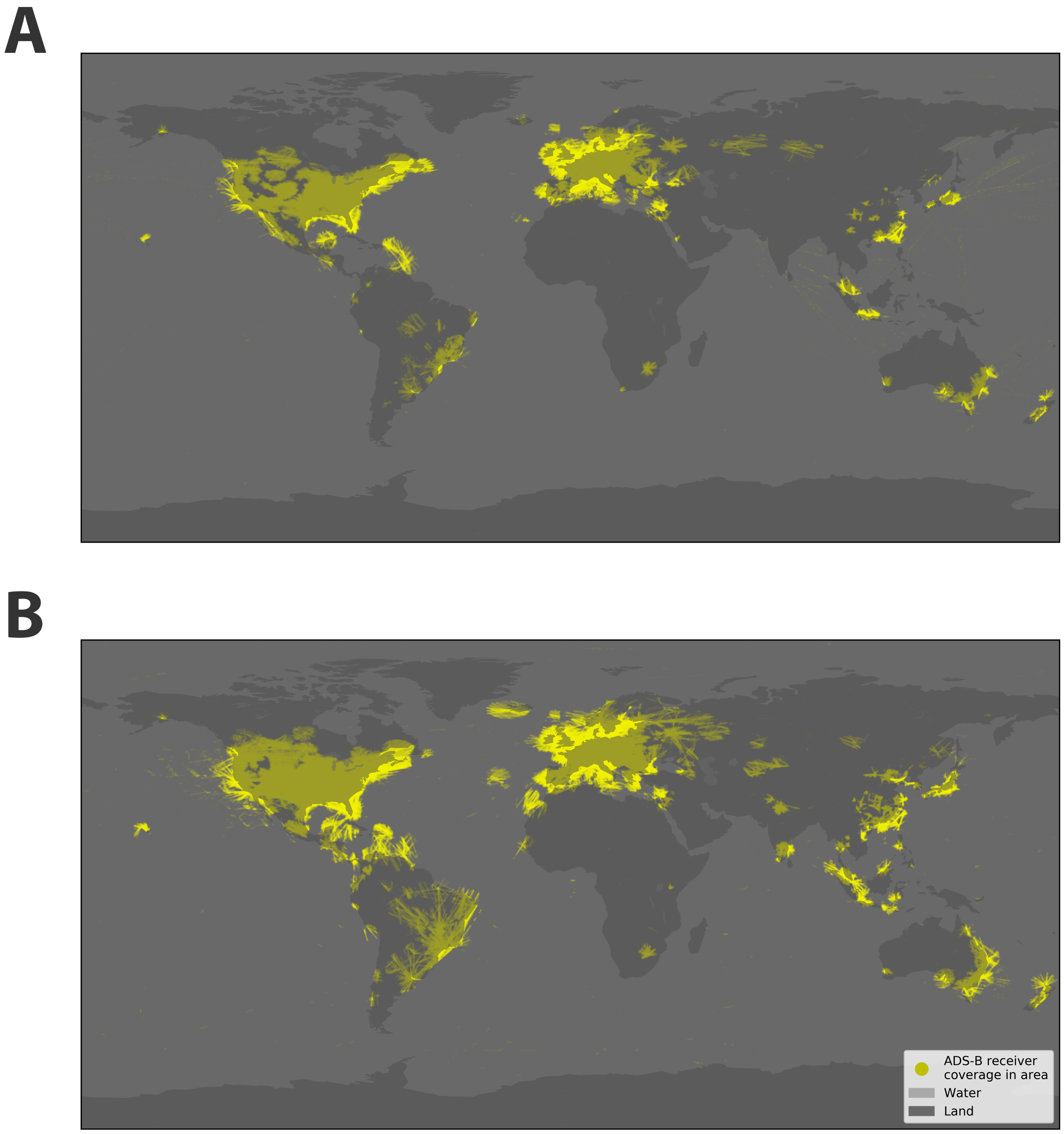

Supplementary Figure S1: This figure depicts coverage over time. An area is highlighted in yellow if an ADS-B message was received from that location on (A) 30th September 2016 or (B) 28th September 2018. The ADS-B Exchange has had good coverage in Western Europe and the coastal USA since launching in June 2016. Coverage has clearly improved across time. This improvement is most noticeable in the central USA, eastern Europe and Brazil. Much of Africa and Asia remains uncovered.

Data cleaning

We use the altitude and timestamp fields from the ADS-B messages to estimate how many flights each aircraft makes per month. Figure S2 shows that, for some aircraft, there are clear errors in altitude data. These errors could reduce the accuracy of our estimates of aircraft activity, so we clean the altitude data using median filtering.

To median filter, we first set a window size $k = 5$. For each altitude observation a_t, we calculate the median of observations in the window $[a_{t-2}, a_{t-1}, a_t, a_{t+1}, a_{t+2}]$. If the altitude is different from the median then we replace it with the median.

To give some numeric examples, suppose we observe the following altitude vector for an aircraft $A_1 = [10000, 10100, 10200, 2000, 10400, 10500, 10600]$. There are 3 points that have enough neighbours to apply the median filter: a_3, a_4, a_5.

- $a_3 = 10200$: neighbours $[10000, 10100, 10200, 2000, 10500]$ has median 10100, so replace it.
- $a_4 = 2000$: neighbours $[10100, 10200, 2000, 10400, 10500]$ has median 10200, so replace it.
- $a_5 = 10400$: neighbours $[10200, 2000, 10400, 10500, 10600$ has median 10400, so do not replace it.

The final vector $A_1^{medfilt} = [10000, 10100, 10100, 10200, 10400, 10500, 10600]$ is a more realistic take-off trajectory. Figure S2 depicts the impact of median filtering on 3 aircraft over a given day. The upper panel is an aircraft with fairly clean data, so filtering changes only 0.8% of observations. The middle and lower panels show aircraft with less clean data, so filtering changes 1.2% and 2.8% of their observations respectively.

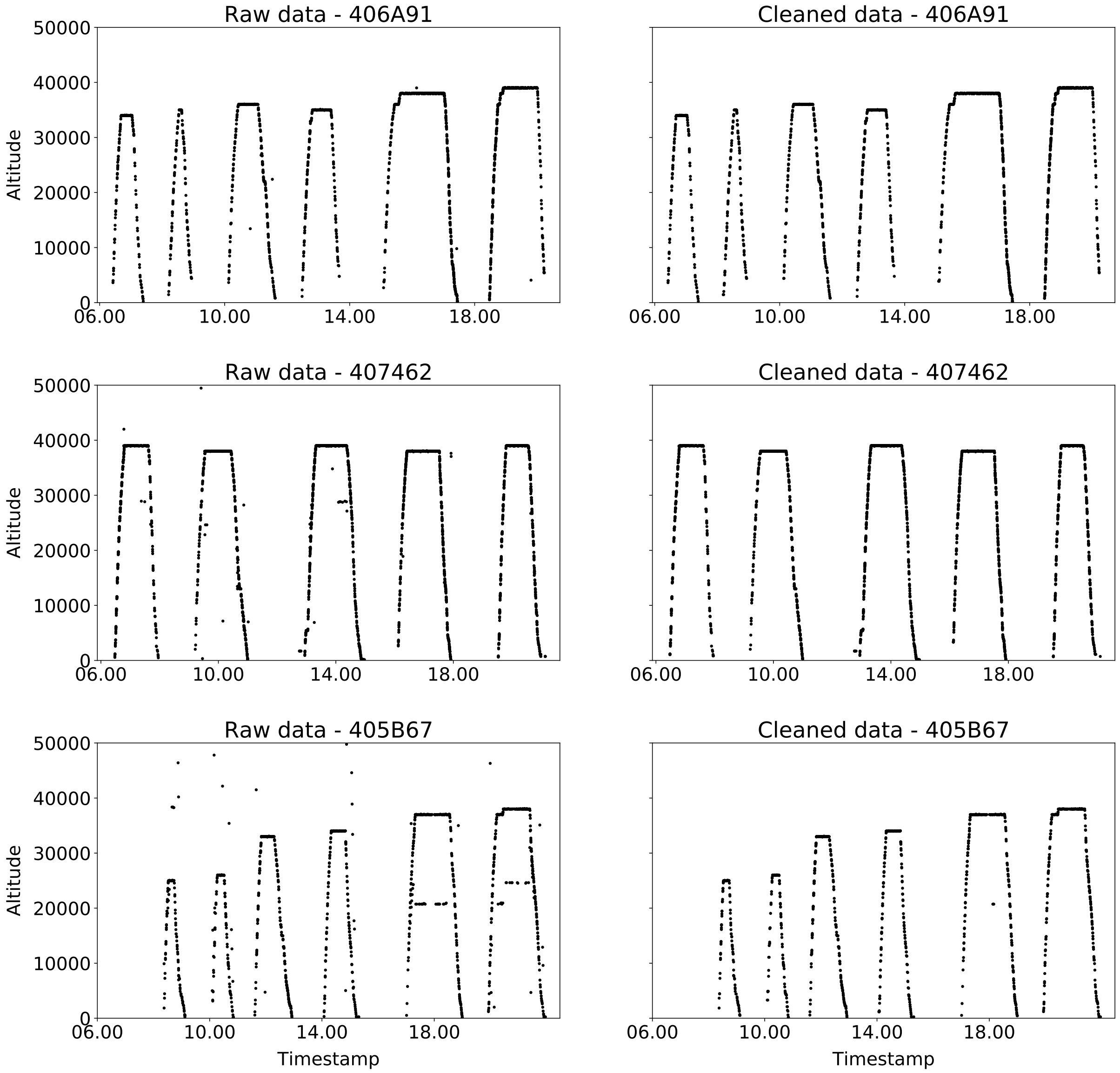

Supplementary Figure S2: Impact of median filtering on aircraft altitude profile. The left panel shows the raw data, and the right panel shows data after median filtering.

Flight-counting algorithm

The raw data contains over 25 billion messages. Initially, there is one row for each message containing, among other fields, the aircraft's latitude, longitude, altitude and timestamp. To estimate monthly airline performance, we do not need such a large volume of data. To facilitate our analysis, we therefore reduce the data to one row for each unique flight. This contains the aircraft's take-off time and location, landing time and location, aircraft ID and the airline.

Counting unique flights from the ADS-B messages is a non-trivial task. Firstly, each aircraft can make multiple flights per day, and there is a lot of variation depending on journey length. We show the distribution of daily flight counts per aircraft in Figure S3.

There is a field in the ADS-B message identifying the aircraft, but no reliable field to identify separate flights. We therefore created an algorithm to identify separate flights from the raw altitude data, henceforth referred to as the *flight-counting algorithm*. The flight-counting algorithm uses the aircraft's altitude to identify the take-off and landing of each flight. It crawls through the time-ordered altitude observations and creates a take-off if the aircraft ascends above a certain threshold. Similarly, it creates a landing if the aircraft descends below a given threshold. The below figures shows how the algorithm identifies take-offs and landings for given aircraft over the course of a day. The counting algorithm reduces the data from having a row for each ADS-B message to a row for each flight. We identify over 67 million flights this way.

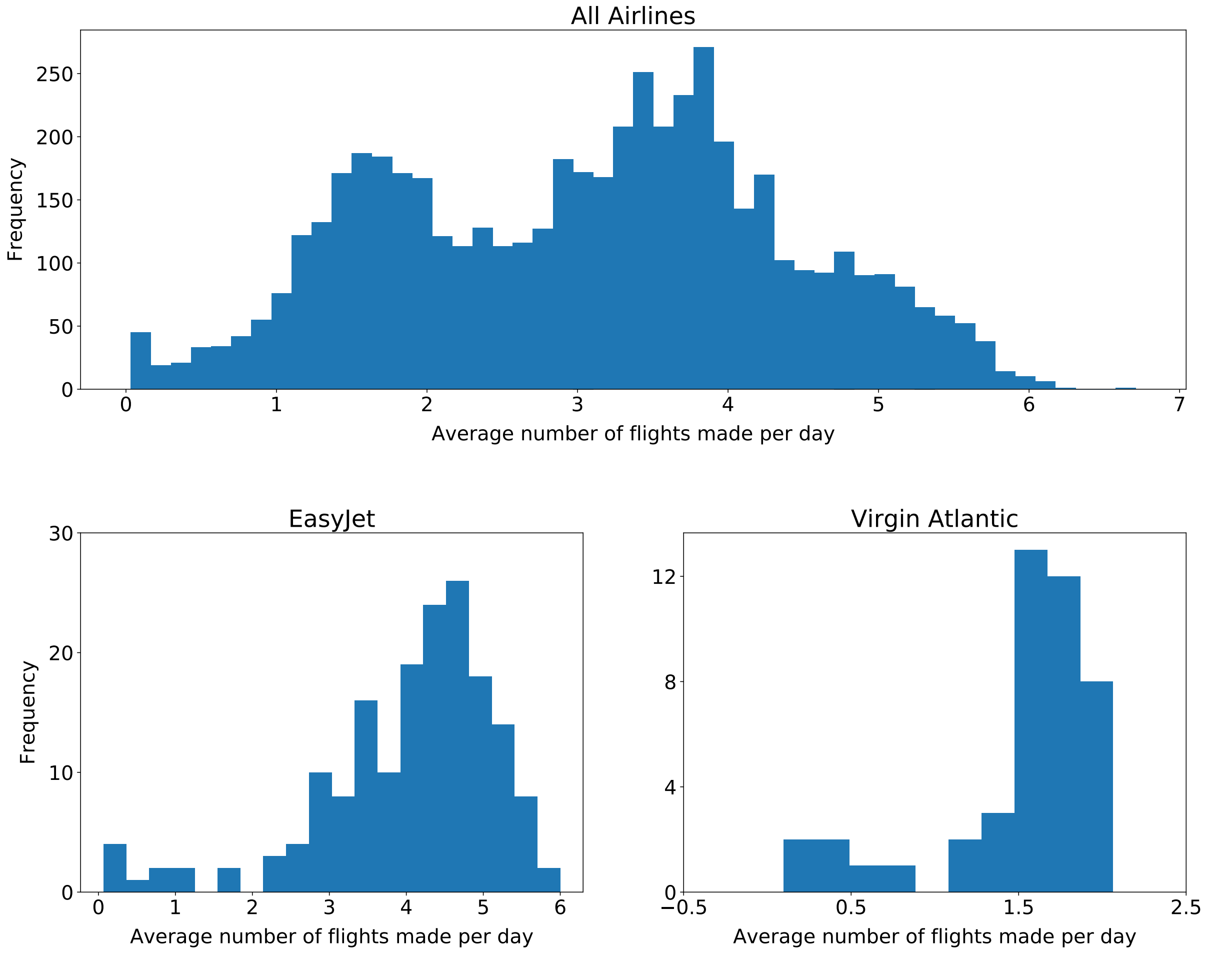

Supplementary Figure S3: Distributions of daily flight counts for each aircraft in September 2018. The upper panel shows that the range is wide: many aircraft make just one flight, but some make up to ten flights. Most of this variation is explained by journey length, which varies systematically by airline. The lower panels show that Easyjet aircraft, which fly short haul, make far more flights on average than Virgin Atlantic, which also fly long haul.

Second, the raw data is not very clean, as shown in Figure S2. This can reduce the flight-counting algorithm's accuracy. Figure S4 depicts the impact of cleaning the altitude data. The left panel shows counts carried out on the raw data, and we can see that they are often wrong. The right panel shows more accurate counts carried out on the cleaned data.

Even after filtering, the altitude data still has occasional errors. To minimise their impact, we introduce some heuristics to the algorithm. If a take-off (landing) is recorded, we stipulate a lag of 30 minutes before the aircraft can land (take-off) again. We select this lag as a reasonable minimum journey time for commercial flights. Figure S5 illustrates how adding the lag heuristic makes the counting algorithm more robust to any residual noise in the data.

Finally, we set the landing altitude at 10,000 feet which is much higher than the take-off altitude of 1,000 feet. This is to minimise the impact of missing data on the algorithm accuracy. Figure S6 shows that higher landing thresholds are much less vulnerable to missing data, although they record landing times slightly too early. We set the take-off threshold to pick up as many take-offs as possible while accounting for the issue that some airports are above sea level. We would not want to record planes that are moving on the ground, while sending ADS-B messages, as take-offs.

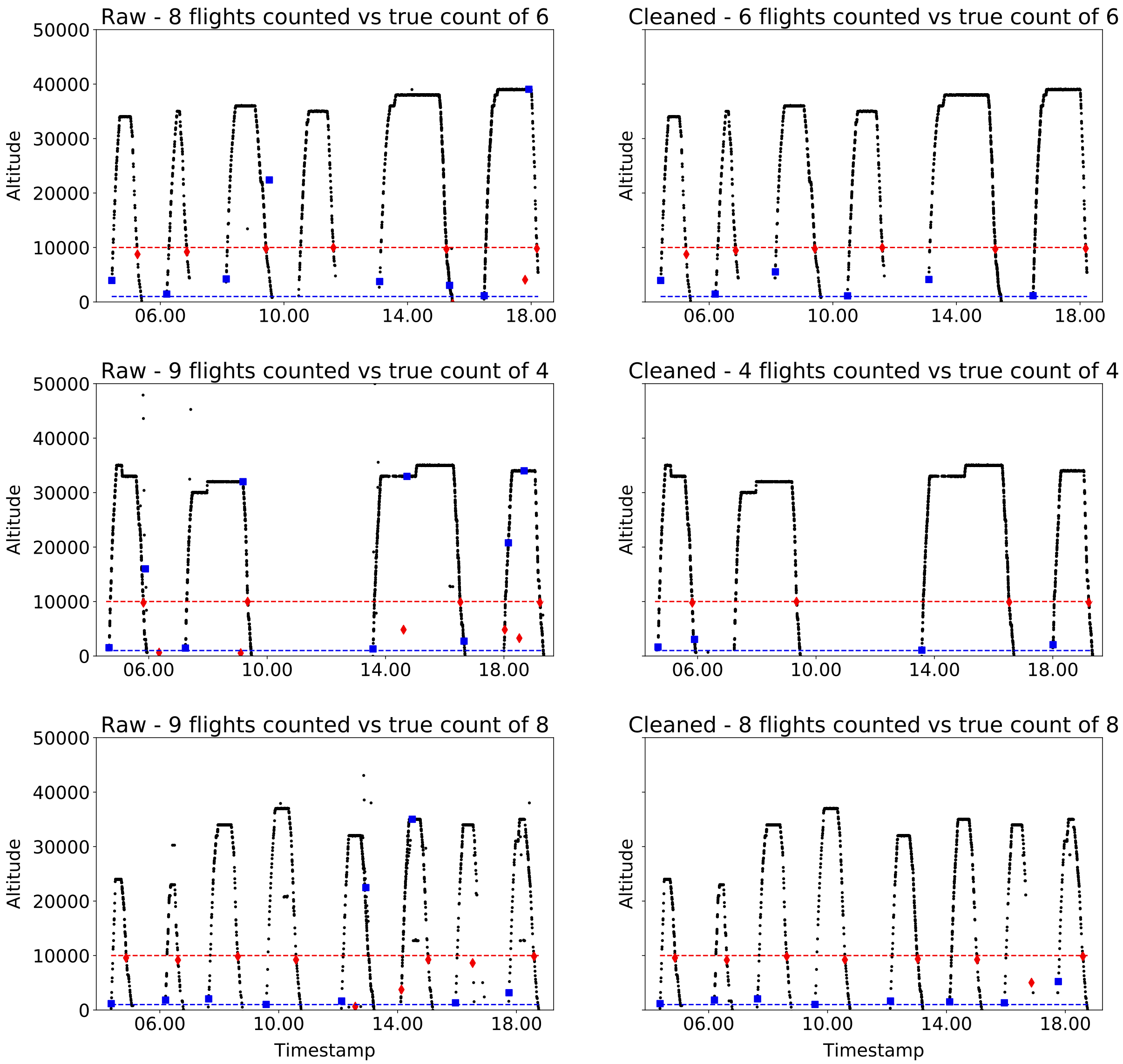

Supplementary Figure S4: The left panel shows the counting algorithm on the raw data for given aircraft over a day. Noise in the data causes the algorithm to identify false positives, so it counts too many flights. The right panel shows the counting algorithm operating on the same data after median filtering. Visual inspection suggests that the algorithm is more accurate after cleaning.

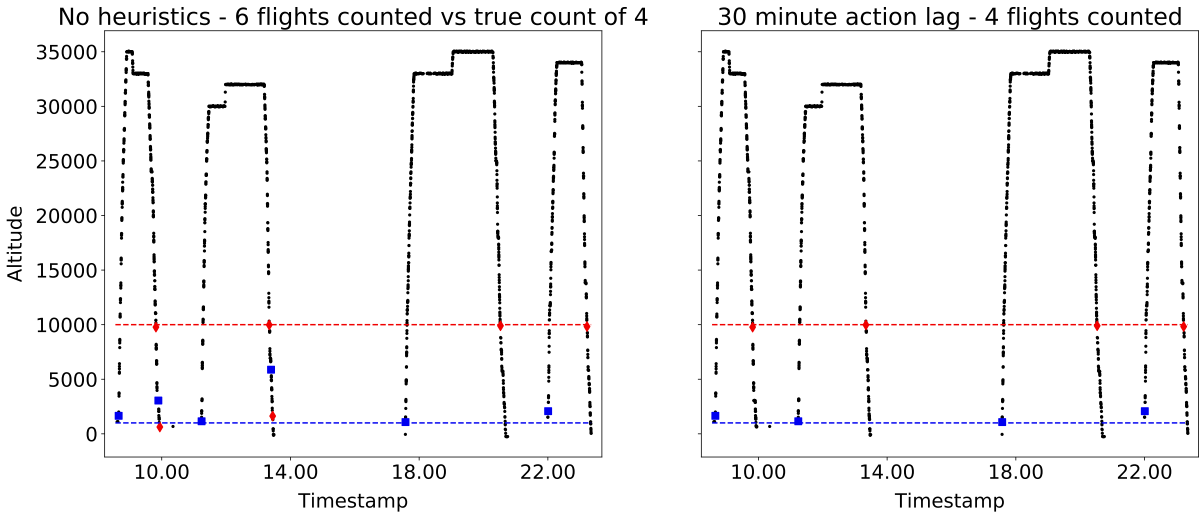

Supplementary Figure S5: This figure demonstrates the importance of enforcing a lag between recording actions for an aircraft. Even after median filtering, there may be residual issues with data quality. The left figure shows these issues can lead to recording of multiple false positives. The right figure shows that enforcing a lag of 30 minutes between actions can help deal with residual data quality issues.

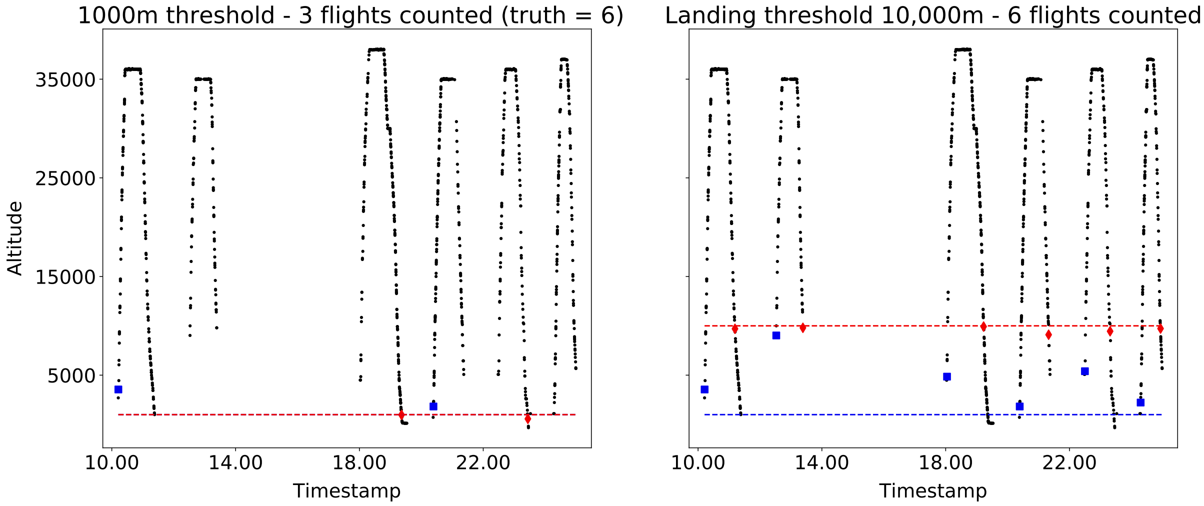

Supplementary Figure S6: This figure demonstrates the importance of setting a landing altitude threshold well above sea level. The left panel shows that a low altitude causes the algorithm to miss several landings, so underestimates the number of flights. The right panel shows that raising the altitude threshold helps correct this, as the algorithm now records six flights.

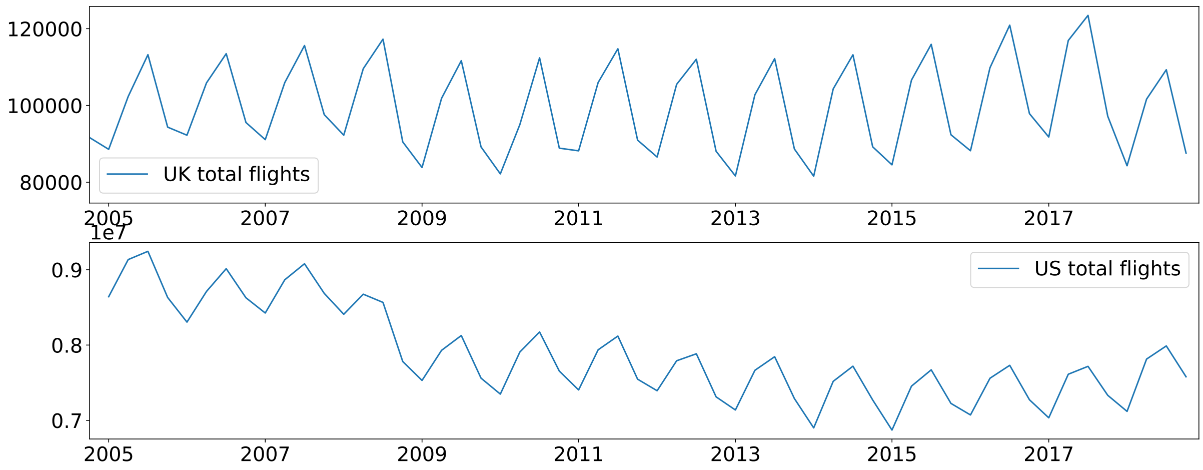

Supplementary Figure S7: This figure depicts the total (across airline) flight volumes series. There is clear seasonality with consistently more flights in summer than winter.

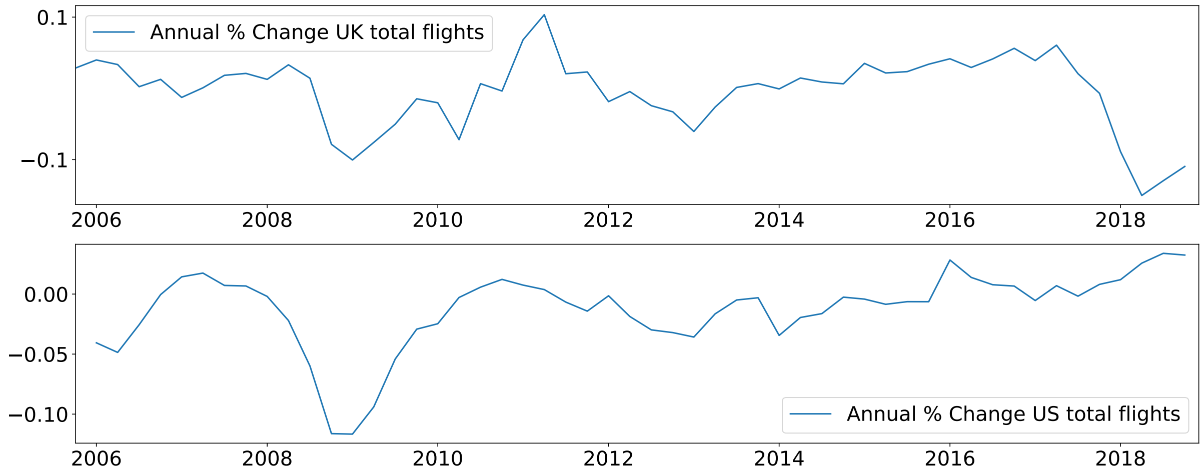

Supplementary Figure S8: Total flights series after conversion to annual percentage changes. No obvious seasonality remains after this conversion.

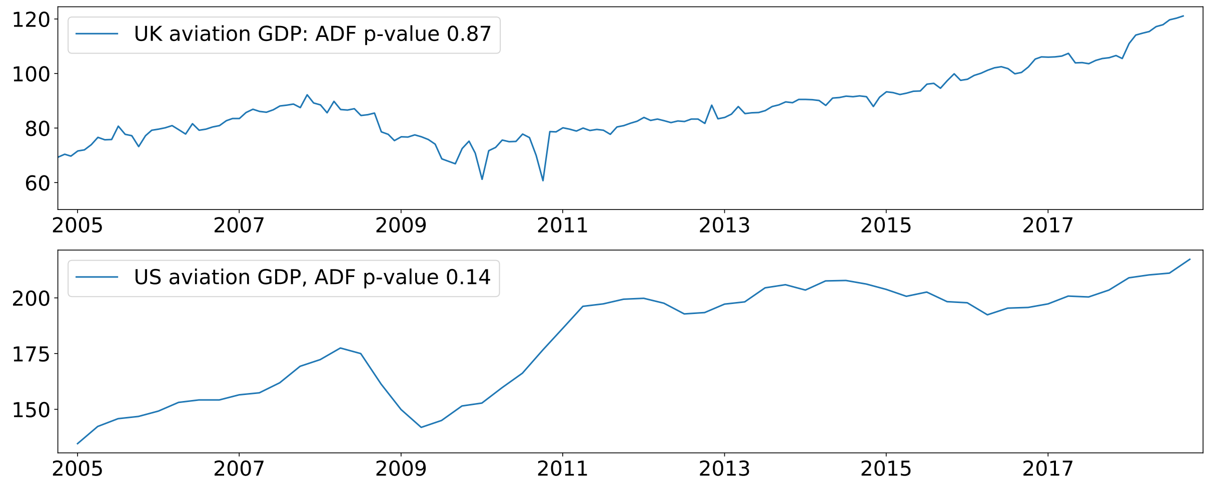

Supplementary Figure S9: This figure shows GDP series. These are both non-stationary, with the Augmented Dickey-Fuller test failing to reject the null hypothesis.

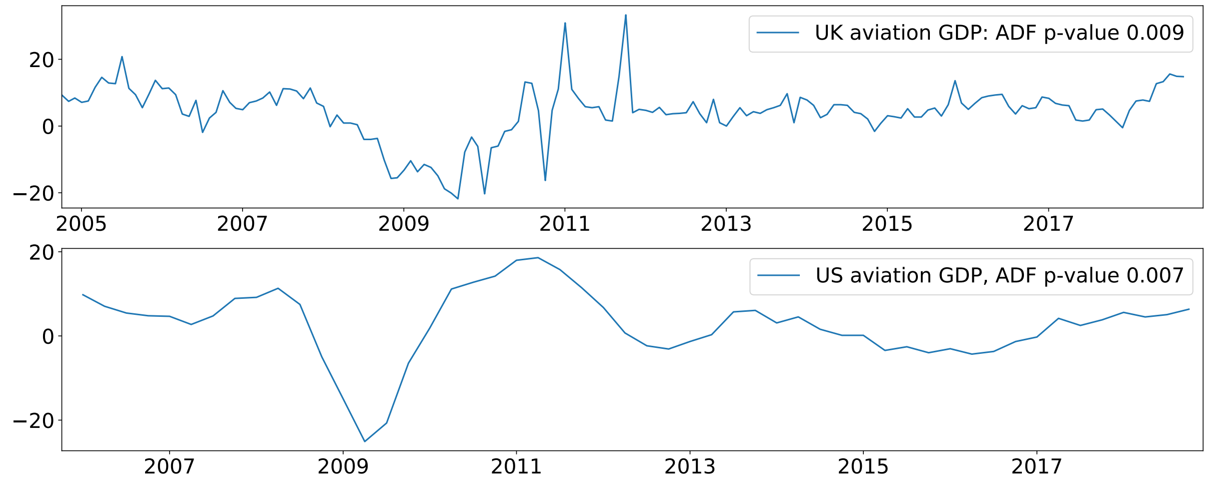

Supplementary Figure S10: This figure shows the GDP series after conversion to annual percentage changes. The null hypothesis of the Augmented Dickey-Fuller test is now rejected for both series (p-values < 0.01).

Supplementary Table S1: Estimating airline performance: in-sample results with different dummy choices. In-sample adjusted R^2 scores from predicting each airline's monthly flights. All models are unpenalised linear regression. The baseline model makes predictions without ADS-B data and the ADS-B score is after adding ADS-B data. Model 1 uses only airline's flights from the previous period as a predictor. Model 2 adds 12 month dummy variables to proxy for seasonality, model 3 adds airline dummy variables, and model 4 uses both.

	UK				US			
Dummies	None	Airlines	Months	All	None	Airlines	Months	All
Baseline R^2	0.06	0.37	0.39	0.66	0.36	0.53	0.61	0.75
ADS-B R^2	0.54	0.82	0.73	0.90	0.56	0.78	0.71	0.89
Number of parameters	2	15	14	27	2	17	14	29

Regularising models for out-of-sample forecasts

We cannot use a random train-test split to assess out-of-sample performance because time series data is not independently and identically distributed (i.i.d.). A random train-test split would put data in the training set that occurs after the data in the testing set. We would therefore use data from the future to fit a model that estimates the past, which would not be a valid measure of out-of-sample accuracy.

Instead, we use adaptive nowcasting to measure out-of-sample accuracy. For each period t in our dataset, we use periods $\in [1, t-1]$ as our training set. The trained model then estimates the flight volumes for each airline in period t. We record the mean absolute error (MAE) across airlines, and that is the test score for period t. Each time we increase t, we re-fit the model to add new training data (which is why we call it "adaptive"). This procedure only uses past data to predict the present, so we know performance is out-of-sample.

The models with airline and time dummies have many parameters. This could lead to over-fitting, which would reduce out-of-sample performance. We therefore regularise our adaptive nowcast models using LASSO regression. Let β be the vector of parameters in a linear forecasting model for T time periods and N airlines:

$$\beta_{LASSO} = min\{\sum_{i=1}^{N}\sum_{t=1}^{T}(y_{i,t} - \beta X_{i,t})^2 + \lambda\|\beta\|\} \tag{1}$$

where $y_{i,t}$ is the flight count for airline i in period t, and $X_{i,t}$ is their feature vector. LASSO applies a linear penalty λ to the magnitude of each coefficient, which punishes more complex models. It also allows automatic variable selection as the linear penalty results in many zero parameters. The weight to penalise complexity is determined by λ. We tune using 5-fold cross-validation across all data from period 1 to $t-1$ to find the optimal values of λ and β. Next we record the tuned model's predictions for period t, and measure the error for each of the

N airlines. The performance score in period t is the MAE across the N airlines. Figure S11 depicts the distribution of nowcast errors over time for both the baseline and ADS-B models.

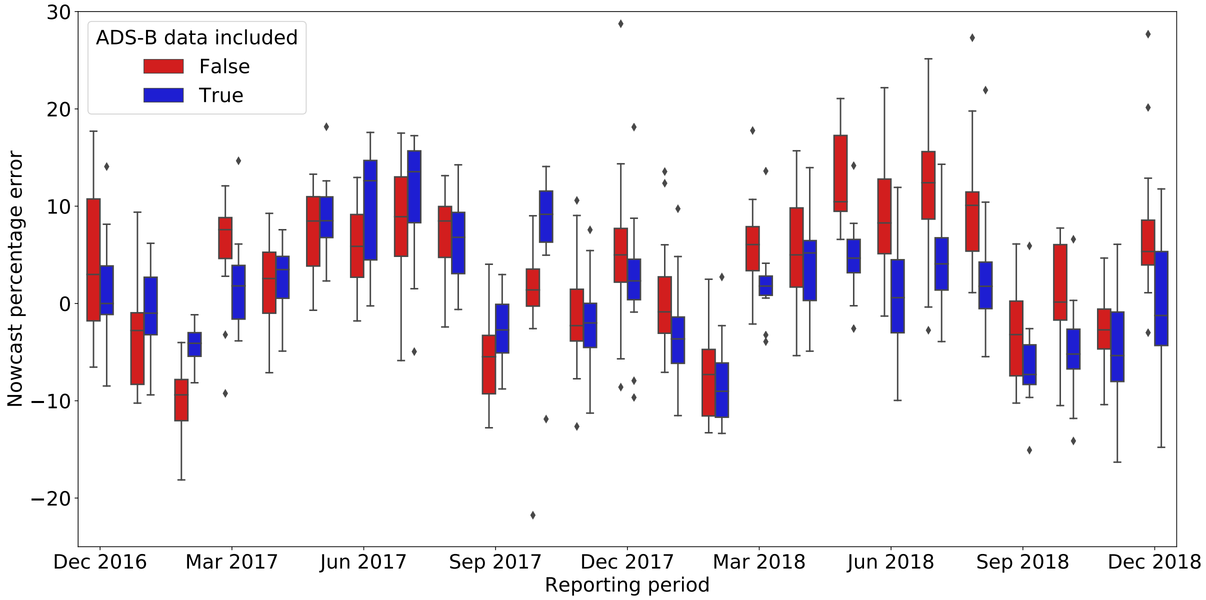

Supplementary Figure S11: Distribution of errors over time.

Different dummy choices for nowcasting airline performance

In the main text, we display results using only airline dummies. We made this choice because fitting month dummies with a short time dimension is difficult. Suppose we are nowcasting for December 2016. Our training data would include July to November 2016. As we had no observations for December, we would not be able to include the month dummies in the predictive model.

Now suppose we are instead nowcasting December 2017. We would have a month of prior airline observations of December to fit the month dummies with, so we would be able to include them. However, they would be very sensitive to any one-off events in December 2016 such as a global network disruption. Furthermore, the model would not be strictly comparable to December 2016 because it would include extra parameters. This would make comparing results across time more difficult.

In this section we show our results are robust to all other choices of dummy variables. Table S2 shows results from these choices. Adding ADS-B data reduces MAE in all specifications. The reductions range from 20% to 26% for the UK, and 11% to 21% for the USA, which is consistent with the results in the main text.

Supplementary Table S2: Adaptive nowcasting results, as measured by MAE, with different dummy variable configurations. The model is LASSO. For each country, column 1 is the simplest model, with only the basic autoregressive predictor. Column 2 adds dummy variables for the airlines, which proxy for airline flight volume growth during the analysis period. Column 3 adds 12 month dummy variables to the model, which proxy for seasonality. Column 4 adds both month and airline dummy variables, which is the most complex specification.

	UK				US			
Baseline MAE	18.8	17.3	17.7	15.2	8.2	7.4	7.3	6.7
ADS-B MAE	14.0	12.2	13.9	12.3	6.9	6.1	6.5	5.3
Dummies	None	Airlines	Months	All	None	Airlines	Months	All

Supplementary Table S3: This table shows adaptive nowcasting results with different training windows. The model is LASSO, with dummy variables for each airline. A fixed training window of w months means we lose the first $w + 1$ months from the sample. Given the short time dimension, our primary setup uses an expanding window instead. Each test period t, we train with all data up to $t - 1$. This minimises data loss, but means later periods are trained on more data than earlier periods. Increasing the training window reduces the size of the test set as the first test period becomes later. Here we report results from a range of fixed training windows from 6 to 18 months. None of them qualitatively differ from the expanding window results in the main text. With a 6 month window, the first period is May 2017, a 12 month window is November 2017 and an 18 month window is May 2018.

	UK			US		
Baseline MAE	21.4	14.7	17.0	7.6	7.3	8.2
Augmented MAE	14.8	11.6	12.0	6.6	5.7	6.2
Window Length	6	12	18	6	12	18

Supplementary Table S4: In the main text, we report summaries of in-sample results using ADS-B directly as a predictor of GDP. This table shows further details of these results. All models are unpenalised linear regression. Baseline is estimation without ADS-B data and ADS-B score is after adding ADS-B data. There is a large boost in R^2 for both the UK and US. However the sample sizes are possibly too small for valid inference. There are only 18 monthly observations for the UK, and 6 quarterly observations for the USA. We cannot construct longer time series as ADS-B data has only been available since July 2016.

Country	UK	USA
Baseline R^2	0.31	0.12
ADS-B R^2	0.55	0.42
Sample size	18	6
Frequency	Monthly	Quarterly

Supplementary Table S5: This table shows the performance split by time period. In the main text, we report that the augmented GDP model performs better relative to the baseline during volatile economic times. The baseline model is relatively strong outside the crisis period, as the autoregressive component is likely a stronger predictor. However during the crisis, when GDP is more volatile, the baseline model is much weaker. The augmented model, which includes real-time flight data as a predictor, becomes relatively stronger. It reduces MAE by 20% for the UK and 46% for the US.

	UK		US	
Time Period	Crisis	Non-crisis	Crisis	Non-crisis
Baseline MAE	8.0	3.2	8.1	2.3
Augmented MAE	6.4	3.3	4.4	2.4
Sample size	48	131	17	25
Frequency	Monthly	Monthly	Quarterly	Quarterly

Supplementary Table S6: In the main text, we claim that our GDP adaptive nowcasting results are robust to varying the length of the training window. The main text shows results from training windows of 60 months for the UK and 8 quarters for the US. This table presents results from varying the training window. For both countries, the second column is the training window length reported in the main text. In the first and third columns for each country, we show how the results change if the training window is increased or decreased by 6 months (2 quarters). Our results are qualitatively unchanged by both shorter and longer training windows, across both the UK and US.

	UK			US		
Train Window	54 months	60 months	66 months	6 quarters	8 quarters	10 quarters
Baseline MAE	4.63	4.54	4.52	4.90	4.65	4.03
Augmented MAE	4.41	4.22	4.13	4.42	3.25	3.10
Sample size	197	191	185	44	42	40
Frequency	Monthly	Monthly	Monthly	Quarterly	Quarterly	Quarterly
